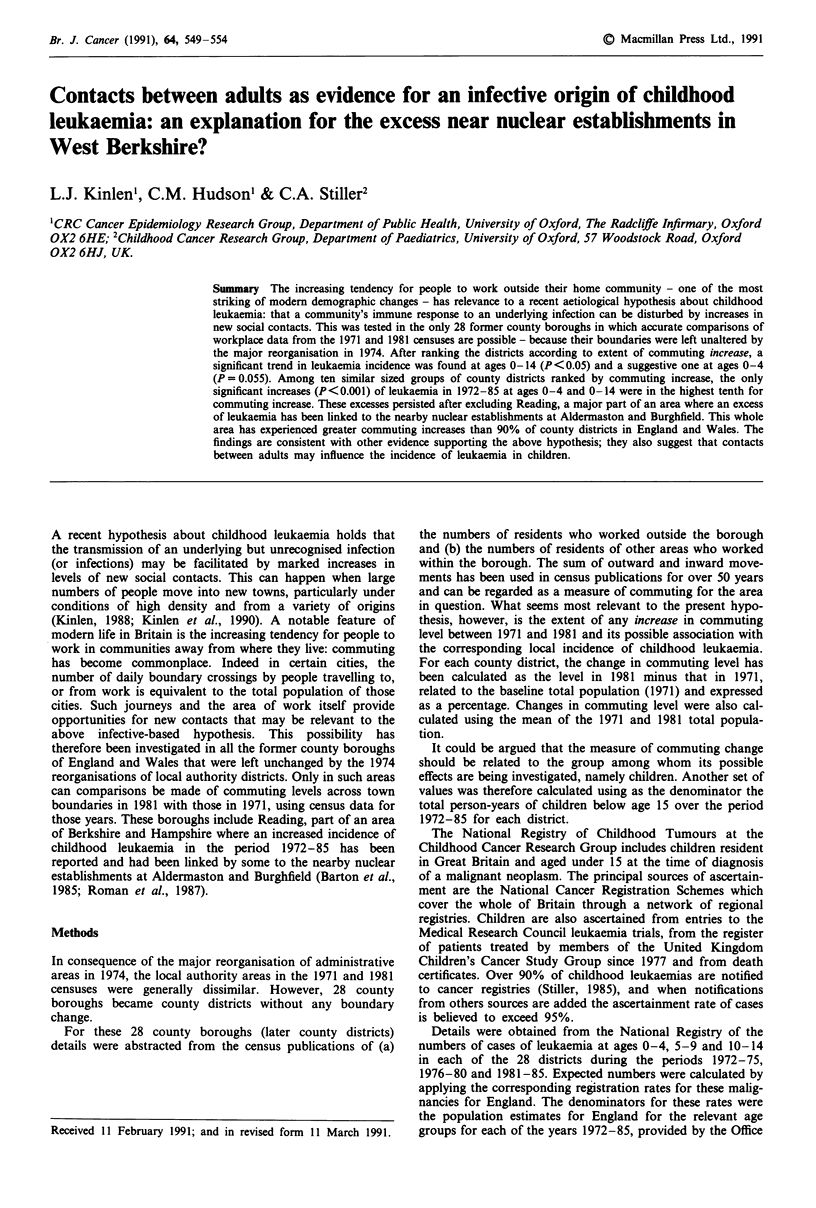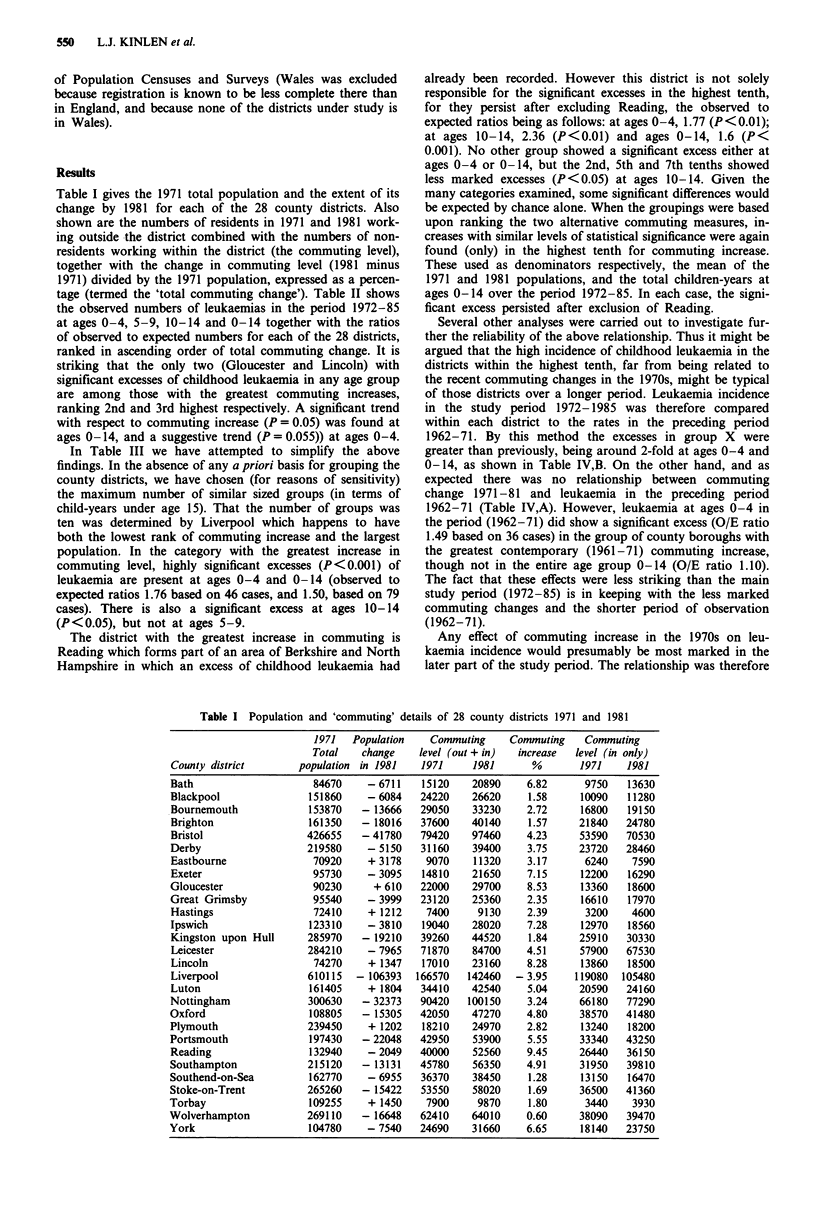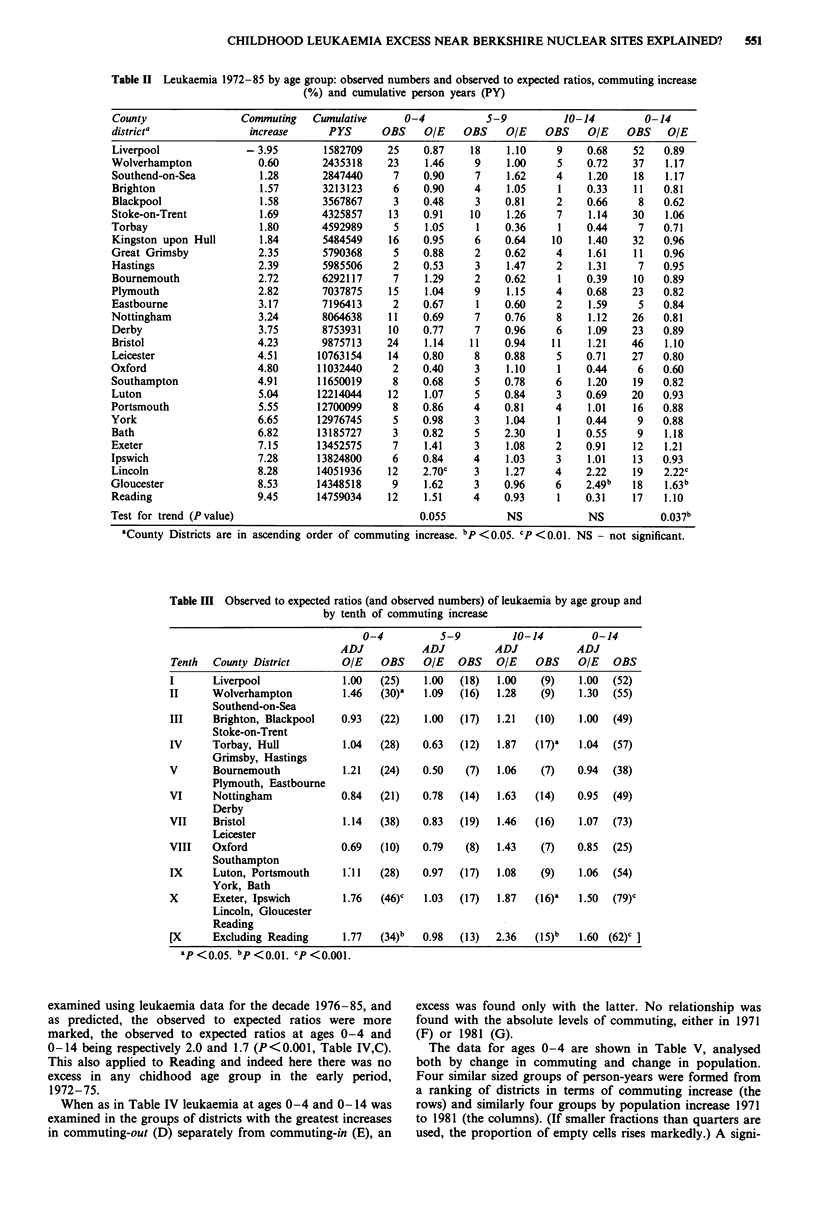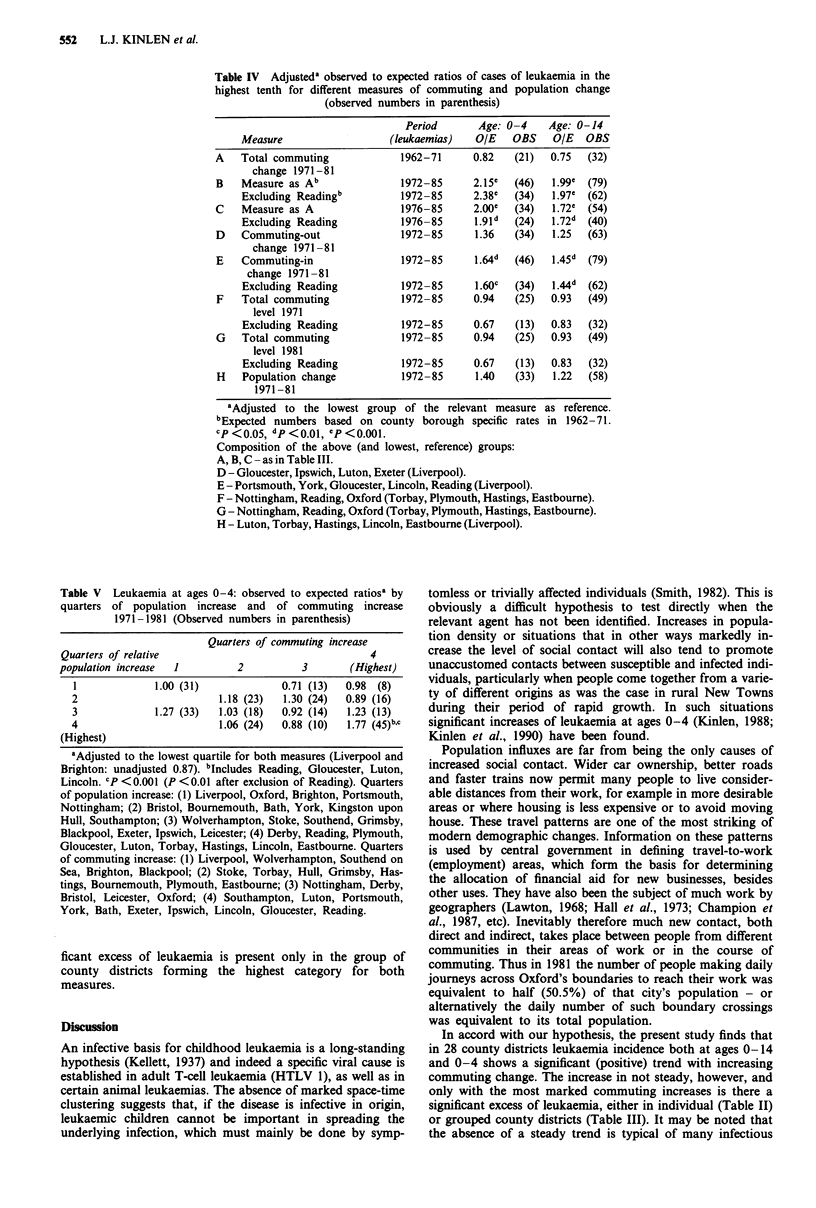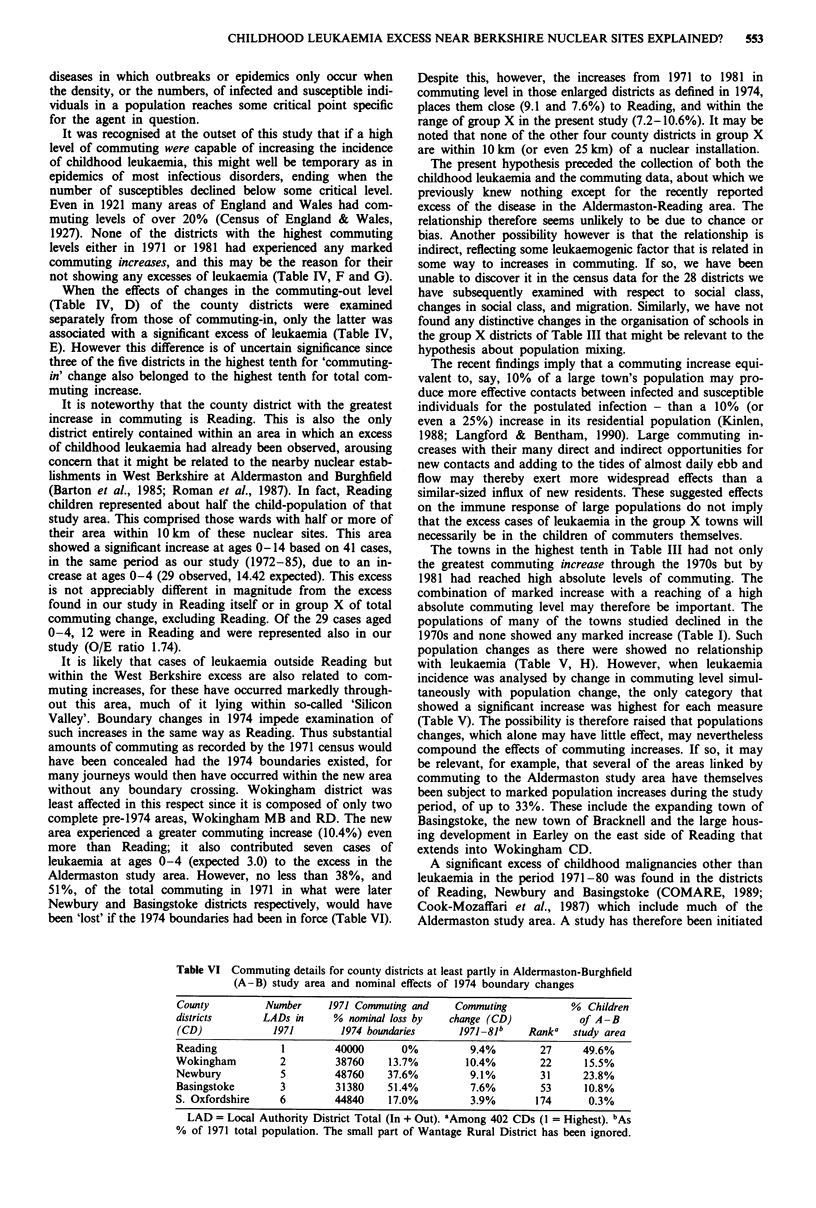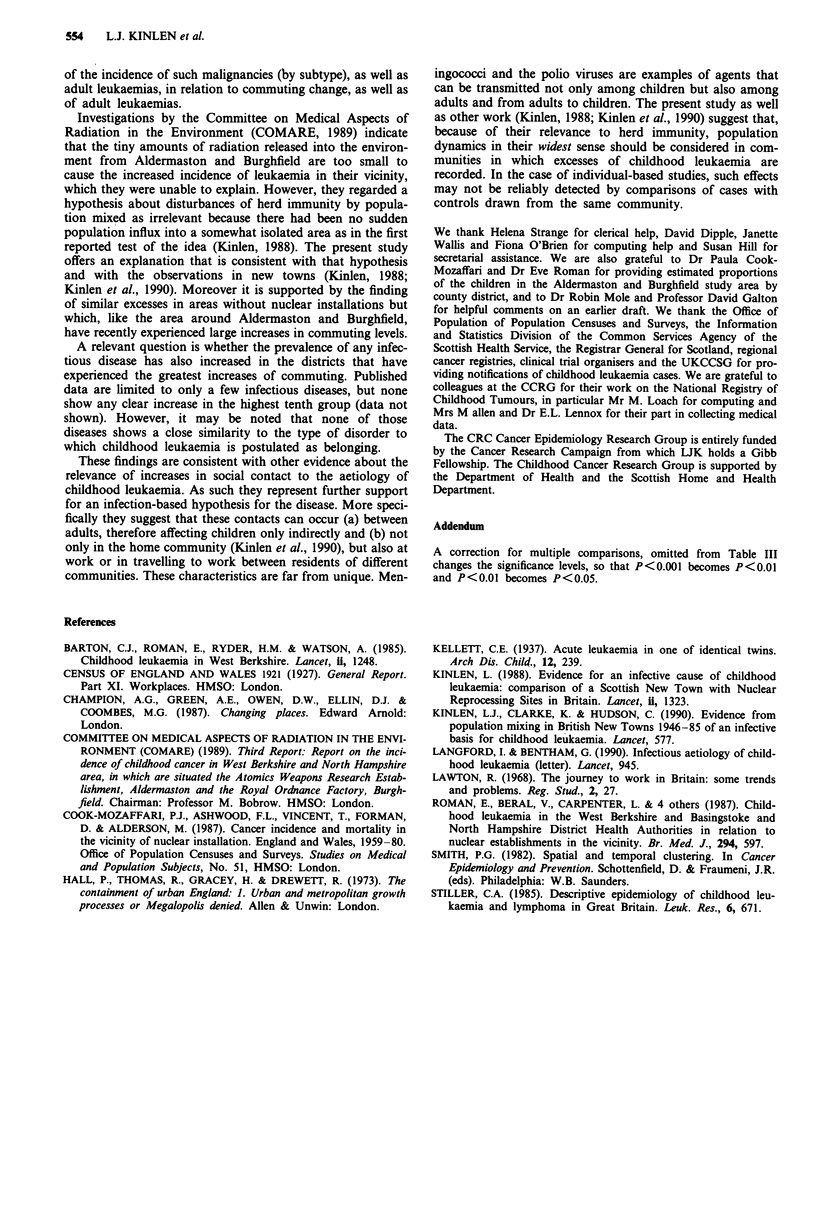# Contacts between adults as evidence for an infective origin of childhood leukaemia: an explanation for the excess near nuclear establishments in west Berkshire?

**DOI:** 10.1038/bjc.1991.348

**Published:** 1991-09

**Authors:** L. J. Kinlen, C. M. Hudson, C. A. Stiller

**Affiliations:** Department of Public Health, University of Oxford, Radcliffe Infirmary, UK.

## Abstract

The increasing tendency for people to work outside their home community--one of the most striking of modern demographic changes--has relevance to a recent aetiological hypothesis about childhood leukaemia: that a community's immune response to an underlying infection can be disturbed by increases in new social contacts. This was tested in the only 28 former county boroughs in which accurate comparisons of workplace data from the 1971 and 1981 censuses are possible--because their boundaries were left unaltered by the major reorganisation in 1974. After ranking the districts according to extent of commuting increase, a significant trend in leukaemia incidence was found at ages 0-14 (P less than 0.05) and a suggestive one at ages 0-4 (P = 0.055). Among ten similar sized groups of county districts ranked by commuting increase, the only significant increases (P less than 0.001) of leukaemia in 1972-85 at ages 0-4 and 0-14 were in the highest tenth for commuting increase. These excesses persisted after excluding Reading, a major part of an area where an excess of leukaemia has been linked to the nearby nuclear establishments at Aldermaston and Burghfield. This whole area has experienced greater commuting increases than 90% of county districts in England and Wales. The findings are consistent with other evidence supporting the above hypothesis; they also suggest that contacts between adults may influence the incidence of leukaemia in children.


Br. J. Cancer (1991), 64, 549–554 © Macmillan Press Ltd., 1991

### Contacts between adults as evidence for an infective origin of childhood leukaemia: an explanation for the excess near nuclear establishments in West Berkshire?

L.J. Kinlen[1], C.M. Hudson[1] & C.A. Stiller[2]

[1]*CRC Cancer Epidemiology Research Group, Department of Public Health, University of Oxford, The Radcliffe Infirmary, Oxford OX2 6HE;* [2]*Childhood Cancer Research Group, Department of Paediatrics, University of Oxford, 57 Woodstock Road, Oxford OX2 6HJ, UK.*

**Summary** The increasing tendency for people to work outside their home community – one of the most striking of modern demographic changes – has relevance to a recent aetiological hypothesis about childhood leukaemia: that a community's immune response to an underlying infection can be disturbed by increases in new social contacts. This was tested in the only 28 former county boroughs in which accurate comparisons of workplace data from the 1971 and 1981 censuses are possible – because their boundaries were left unaltered by the major reorganisation in 1974. After ranking the districts according to extent of commuting *increase*, a significant trend in leukaemia incidence was found at ages 0–14 ($P < 0.05$) and a suggestive one at ages 0–4 ($P = 0.055$). Among ten similar sized groups of county districts ranked by commuting increase, the only significant increases ($P < 0.001$) of leukaemia in 1972–85 at ages 0–4 and 0–14 were in the highest tenth for commuting increase. These excesses persisted after excluding Reading, a major part of an area where an excess of leukaemia has been linked to the nearby nuclear establishments at Aldermaston and Burghfield. This whole area has experienced greater commuting increases than 90% of county districts in England and Wales. The findings are consistent with other evidence supporting the above hypothesis; they also suggest that contacts between adults may influence the incidence of leukaemia in children.

A recent hypothesis about childhood leukaemia holds that the transmission of an underlying but unrecognised infection (or infections) may be facilitated by marked increases in levels of new social contacts. This can happen when large numbers of people move into new towns, particularly under conditions of high density and from a variety of origins (Kinlen, 1988; Kinlen *et al.*, 1990). A notable feature of modern life in Britain is the increasing tendency for people to work in communities away from where they live: commuting has become commonplace. Indeed in certain cities, the number of daily boundary crossings by people travelling to, or from work is equivalent to the total population of those cities. Such journeys and the area of work itself provide opportunities for new contacts that may be relevant to the above infective-based hypothesis. This possibility has therefore been investigated in all the former county boroughs of England and Wales that were left unchanged by the 1974 reorganisations of local authority districts. Only in such areas can comparisons be made of commuting levels across town boundaries in 1981 with those in 1971, using census data for those years. These boroughs include Reading, part of an area of Berkshire and Hampshire where an increased incidence of childhood leukaemia in the period 1972–85 has been reported and had been linked by some to the nearby nuclear establishments at Aldermaston and Burghfield (Barton *et al.*, 1985; Roman *et al.*, 1987).

### Methods

In consequence of the major reorganisation of administrative areas in 1974, the local authority areas in the 1971 and 1981 censuses were generally dissimilar. However, 28 county boroughs became county districts without any boundary change.

For these 28 county boroughs (later county districts) details were abstracted from the census publications of (a) the numbers of residents who worked outside the borough and (b) the numbers of residents of other areas who worked within the borough. The sum of outward and inward movements has been used in census publications for over 50 years and can be regarded as a measure of commuting for the area in question. What seems most relevant to the present hypothesis, however, is the extent of any *increase* in commuting level between 1971 and 1981 and its possible association with the corresponding local incidence of childhood leukaemia. For each county district, the change in commuting level has been calculated as the level in 1981 minus that in 1971, related to the baseline total population (1971) and expressed as a percentage. Changes in commuting level were also calculated using the mean of the 1971 and 1981 total population.

It could be argued that the measure of commuting change should be related to the group among whom its possible effects are being investigated, namely children. Another set of values was therefore calculated using as the denominator the total person-years of children below age 15 over the period 1972–85 for each district.

The National Registry of Childhood Tumours at the Childhood Cancer Research Group includes children resident in Great Britain and aged under 15 at the time of diagnosis of a malignant neoplasm. The principal sources of ascertainment are the National Cancer Registration Schemes which cover the whole of Britain through a network of regional registries. Children are also ascertained from entries to the Medical Research Council leukaemia trials, from the register of patients treated by members of the United Kingdom Children's Cancer Study Group since 1977 and from death certificates. Over 90% of childhood leukaemias are notified to cancer registries (Stiller, 1985), and when notifications from others sources are added the ascertainment rate of cases is believed to exceed 95%.

Details were obtained from the National Registry of the numbers of cases of leukaemia at ages 0–4, 5–9 and 10–14 in each of the 28 districts during the periods 1972–75, 1976–80 and 1981–85. Expected numbers were calculated by applying the corresponding registration rates for these malignancies for England. The denominators for these rates were the population estimates for England for the relevant age groups for each of the years 1972–85, provided by the Office

Received 11 February 1991; and in revised form 11 March 1991.

of Population Censuses and Surveys (Wales was excluded because registration is known to be less complete there than in England, and because none of the districts under study is in Wales).

### Results

Table I gives the 1971 total population and the extent of its change by 1981 for each of the 28 county districts. Also shown are the numbers of residents in 1971 and 1981 working outside the district combined with the numbers of non-residents working within the district (the commuting level), together with the change in commuting level (1981 minus 1971) divided by the 1971 population, expressed as a percentage (termed the 'total commuting change'). Table II shows the observed numbers of leukaemias in the period 1972–85 at ages 0–4, 5–9, 10–14 and 0–14 together with the ratios of observed to expected numbers for each of the 28 districts, ranked in ascending order of total commuting change. It is striking that the only two (Gloucester and Lincoln) with significant excesses of childhood leukaemia in any age group are among those with the greatest commuting increases, ranking 2nd and 3rd highest respectively. A significant trend with respect to commuting increase ($P = 0.05$) was found at ages 0–14, and a suggestive trend ($P = 0.055$)) at ages 0–4.

In Table III we have attempted to simplify the above findings. In the absence of any *a priori* basis for grouping the county districts, we have chosen (for reasons of sensitivity) the maximum number of similar sized groups (in terms of child-years under age 15). That the number of groups was ten was determined by Liverpool which happens to have both the lowest rank of commuting increase and the largest population. In the category with the greatest increase in commuting level, highly significant excesses ($P < 0.001$) of leukaemia are present at ages 0–4 and 0–14 (observed to expected ratios 1.76 based on 46 cases, and 1.50, based on 79 cases). There is also a significant excess at ages 10–14 ($P < 0.05$), but not at ages 5–9.

The district with the greatest increase in commuting is Reading which forms part of an area of Berkshire and North Hampshire in which an excess of childhood leukaemia had already been recorded. However this district is not solely responsible for the significant excesses in the highest tenth, for they persist after excluding Reading, the observed to expected ratios being as follows: at ages 0–4, 1.77 ($P < 0.01$); at ages 10–14, 2.36 ($P < 0.01$) and ages 0–14, 1.6 ($P < 0.001$). No other group showed a significant excess either at ages 0–4 or 0–14, but the 2nd, 5th and 7th tenths showed less marked excesses ($P < 0.05$) at ages 10–14. Given the many categories examined, some significant differences would be expected by chance alone. When the groupings were based upon ranking the two alternative commuting measures, increases with similar levels of statistical significance were again found (only) in the highest tenth for commuting increase. These used as denominators respectively, the mean of the 1971 and 1981 populations, and the total children-years at ages 0–14 over the period 1972–85. In each case, the significant excess persisted after exclusion of Reading.

Several other analyses were carried out to investigate further the reliability of the above relationship. Thus it might be argued that the high incidence of childhood leukaemia in the districts within the highest tenth, far from being related to the recent commuting changes in the 1970s, might be typical of those districts over a longer period. Leukaemia incidence in the study period 1972–1985 was therefore compared within each district to the rates in the preceding period 1962–71. By this method the excesses in group X were greater than previously, being around 2-fold at ages 0–4 and 0–14, as shown in Table IV,B. On the other hand, and as expected there was no relationship between commuting change 1971–81 and leukaemia in the preceding period 1962–71 (Table IV,A). However, leukaemia at ages 0–4 in the period (1962–71) did show a significant excess (O/E ratio 1.49 based on 36 cases) in the group of county boroughs with the greatest contemporary (1961–71) commuting increase, though not in the entire age group 0–14 (O/E ratio 1.10). The fact that these effects were less striking than the main study period (1972–85) is in keeping with the less marked commuting changes and the shorter period of observation (1962–71).

Any effect of commuting increase in the 1970s on leukaemia incidence would presumably be most marked in the later part of the study period. The relationship was therefore

**Table I** Population and 'commuting' details of 28 county districts 1971 and 1981

| *County district* | *1971 Total population* | *Population change in 1981* | *Commuting level (out + in) 1971* | *1981* | *Commuting increase %* | *Commuting level (in only) 1971* | *1981* |
|---|---|---|---|---|---|---|---|
| Bath | 84670 | − 6711 | 15120 | 20890 | 6.82 | 9750 | 13630 |
| Blackpool | 151860 | − 6084 | 24220 | 26620 | 1.58 | 10090 | 11280 |
| Bournemouth | 153870 | − 13666 | 29050 | 33230 | 2.72 | 16800 | 19150 |
| Brighton | 161350 | − 18016 | 37600 | 40140 | 1.57 | 21840 | 24780 |
| Bristol | 426655 | − 41780 | 79420 | 97460 | 4.23 | 53590 | 70530 |
| Derby | 219580 | − 5150 | 31160 | 39400 | 3.75 | 23720 | 28460 |
| Eastbourne | 70920 | + 3178 | 9070 | 11320 | 3.17 | 6240 | 7590 |
| Exeter | 95730 | − 3095 | 14810 | 21650 | 7.15 | 12200 | 16290 |
| Gloucester | 90230 | + 610 | 22000 | 29700 | 8.53 | 13360 | 18600 |
| Great Grimsby | 95540 | − 3999 | 23120 | 25360 | 2.35 | 16610 | 17970 |
| Hastings | 72410 | + 1212 | 7400 | 9130 | 2.39 | 3200 | 4600 |
| Ipswich | 123310 | − 3810 | 19040 | 28020 | 7.28 | 12970 | 18560 |
| Kingston upon Hull | 285970 | − 19210 | 39260 | 44520 | 1.84 | 25910 | 30330 |
| Leicester | 284210 | − 7965 | 71870 | 84700 | 4.51 | 57900 | 67530 |
| Lincoln | 74270 | + 1347 | 17010 | 23160 | 8.28 | 13860 | 18500 |
| Liverpool | 610115 | − 106393 | 166570 | 142460 | − 3.95 | 119080 | 105480 |
| Luton | 161405 | + 1804 | 34410 | 42540 | 5.04 | 20590 | 24160 |
| Nottingham | 300630 | − 32373 | 90420 | 100150 | 3.24 | 66180 | 77290 |
| Oxford | 108805 | − 15305 | 42050 | 47270 | 4.80 | 38570 | 41480 |
| Plymouth | 239450 | + 1202 | 18210 | 24970 | 2.82 | 13240 | 18200 |
| Portsmouth | 197430 | − 22048 | 42950 | 53900 | 5.55 | 33340 | 43250 |
| Reading | 132940 | − 2049 | 40000 | 52560 | 9.45 | 26440 | 36150 |
| Southampton | 215120 | − 13131 | 45780 | 56350 | 4.91 | 31950 | 39810 |
| Southend-on-Sea | 162770 | − 6955 | 36370 | 38450 | 1.28 | 13150 | 16470 |
| Stoke-on-Trent | 265260 | − 15422 | 53550 | 58020 | 1.69 | 36500 | 41360 |
| Torbay | 109255 | + 1450 | 7900 | 9870 | 1.80 | 3440 | 3930 |
| Wolverhampton | 269110 | − 16648 | 62410 | 64010 | 0.60 | 38090 | 39470 |
| York | 104780 | − 7540 | 24690 | 31660 | 6.65 | 18140 | 23750 |

**Table II** Leukaemia 1972–85 by age group: observed numbers and observed to expected ratios, commuting increase (%) and cumulative person years (PY)

| *County district*[a] | *Commuting increase* | *Cumulative PYS* | *0–4 OBS* | *0–4 O/E* | *5–9 OBS* | *5–9 O/E* | *10–14 OBS* | *10–14 O/E* | *0–14 OBS* | *0–14 O/E* |
|---|---|---|---|---|---|---|---|---|---|---|
| Liverpool | – 3.95 | 1582709 | 25 | 0.87 | 18 | 1.10 | 9 | 0.68 | 52 | 0.89 |
| Wolverhampton | 0.60 | 2435318 | 23 | 1.46 | 9 | 1.00 | 5 | 0.72 | 37 | 1.17 |
| Southend-on-Sea | 1.28 | 2847440 | 7 | 0.90 | 7 | 1.62 | 4 | 1.20 | 18 | 1.17 |
| Brighton | 1.57 | 3213123 | 6 | 0.90 | 4 | 1.05 | 1 | 0.33 | 11 | 0.81 |
| Blackpool | 1.58 | 3567867 | 3 | 0.48 | 3 | 0.81 | 2 | 0.66 | 8 | 0.62 |
| Stoke-on-Trent | 1.69 | 4325857 | 13 | 0.91 | 10 | 1.26 | 7 | 1.14 | 30 | 1.06 |
| Torbay | 1.80 | 4592989 | 5 | 1.05 | 1 | 0.36 | 1 | 0.44 | 7 | 0.71 |
| Kingston upon Hull | 1.84 | 5484549 | 16 | 0.95 | 6 | 0.64 | 10 | 1.40 | 32 | 0.96 |
| Great Grimsby | 2.35 | 5790368 | 5 | 0.88 | 2 | 0.62 | 4 | 1.61 | 11 | 0.96 |
| Hastings | 2.39 | 5985506 | 2 | 0.53 | 3 | 1.47 | 2 | 1.31 | 7 | 0.95 |
| Bournemouth | 2.72 | 6292117 | 7 | 1.29 | 2 | 0.62 | 1 | 0.39 | 10 | 0.89 |
| Plymouth | 2.82 | 7037875 | 15 | 1.04 | 9 | 1.15 | 4 | 0.68 | 23 | 0.82 |
| Eastbourne | 3.17 | 7196413 | 2 | 0.67 | 1 | 0.60 | 2 | 1.59 | 5 | 0.84 |
| Nottingham | 3.24 | 8064638 | 11 | 0.69 | 7 | 0.76 | 8 | 1.12 | 26 | 0.81 |
| Derby | 3.75 | 8753931 | 10 | 0.77 | 7 | 0.96 | 6 | 1.09 | 23 | 0.89 |
| Bristol | 4.23 | 9875713 | 24 | 1.14 | 11 | 0.94 | 11 | 1.21 | 46 | 1.10 |
| Leicester | 4.51 | 10763154 | 14 | 0.80 | 8 | 0.88 | 5 | 0.71 | 27 | 0.80 |
| Oxford | 4.80 | 11032440 | 2 | 0.40 | 3 | 1.10 | 1 | 0.44 | 6 | 0.60 |
| Southampton | 4.91 | 11650019 | 8 | 0.68 | 5 | 0.78 | 6 | 1.20 | 19 | 0.82 |
| Luton | 5.04 | 12214044 | 12 | 1.07 | 5 | 0.84 | 3 | 0.69 | 20 | 0.93 |
| Portsmouth | 5.55 | 12700099 | 8 | 0.86 | 4 | 0.81 | 4 | 1.01 | 16 | 0.88 |
| York | 6.65 | 12976745 | 5 | 0.98 | 3 | 1.04 | 1 | 0.44 | 9 | 0.88 |
| Bath | 6.82 | 13185727 | 3 | 0.82 | 5 | 2.30 | 1 | 0.55 | 9 | 1.18 |
| Exeter | 7.15 | 13452575 | 7 | 1.41 | 3 | 1.08 | 2 | 0.91 | 12 | 1.21 |
| Ipswich | 7.28 | 13824800 | 6 | 0.84 | 4 | 1.03 | 3 | 1.01 | 13 | 0.93 |
| Lincoln | 8.28 | 14051936 | 12 | 2.70[c] | 3 | 1.27 | 4 | 2.22 | 19 | 2.22[c] |
| Gloucester | 8.53 | 14348518 | 9 | 1.62 | 3 | 0.96 | 6 | 2.49[b] | 18 | 1.63[b] |
| Reading | 9.45 | 14759034 | 12 | 1.51 | 4 | 0.93 | 1 | 0.31 | 17 | 1.10 |
| Test for trend (*P* value) | | | | 0.055 | | NS | | NS | | 0.037[b] |

[a]County Districts are in ascending order of commuting increase. [b]$P < 0.05$. [c]$P < 0.01$. NS – not significant.

**Table III** Observed to expected ratios (and observed numbers) of leukaemia by age group and by tenth of commuting increase

| *Tenth* | *County District* | *0–4 ADJ O/E* | *0–4 OBS* | *5–9 ADJ O/E* | *5–9 OBS* | *10–14 ADJ O/E* | *10–14 OBS* | *0–14 ADJ O/E* | *0–14 OBS* |
|---|---|---|---|---|---|---|---|---|---|
| I | Liverpool | 1.00 | (25) | 1.00 | (18) | 1.00 | (9) | 1.00 | (52) |
| II | Wolverhampton Southend-on-Sea | 1.46 | (30)[a] | 1.09 | (16) | 1.28 | (9) | 1.30 | (55) |
| III | Brighton, Blackpool Stoke-on-Trent | 0.93 | (22) | 1.00 | (17) | 1.21 | (10) | 1.00 | (49) |
| IV | Torbay, Hull Grimsby, Hastings | 1.04 | (28) | 0.63 | (12) | 1.87 | (17)[a] | 1.04 | (57) |
| V | Bournemouth Plymouth, Eastbourne | 1.21 | (24) | 0.50 | (7) | 1.06 | (7) | 0.94 | (38) |
| VI | Nottingham Derby | 0.84 | (21) | 0.78 | (14) | 1.63 | (14) | 0.95 | (49) |
| VII | Bristol Leicester | 1.14 | (38) | 0.83 | (19) | 1.46 | (16) | 1.07 | (73) |
| VIII | Oxford Southampton | 0.69 | (10) | 0.79 | (8) | 1.43 | (7) | 0.85 | (25) |
| IX | Luton, Portsmouth York, Bath | 1.11 | (28) | 0.97 | (17) | 1.08 | (9) | 1.06 | (54) |
| X | Exeter, Ipswich Lincoln, Gloucester Reading | 1.76 | (46)[c] | 1.03 | (17) | 1.87 | (16)[a] | 1.50 | (79)[c] |
| [X | Excluding Reading | 1.77 | (34)[b] | 0.98 | (13) | 2.36 | (15)[b] | 1.60 | (62)[c] ] |

[a]$P < 0.05$. [b]$P < 0.01$. [c]$P < 0.001$.

examined using leukaemia data for the decade 1976–85, and as predicted, the observed to expected ratios were more marked, the observed to expected ratios at ages 0–4 and 0–14 being respectively 2.0 and 1.7 ($P < 0.001$, Table IV,C). This also applied to Reading and indeed here there was no excess in any chidhood age group in the early period, 1972–75.

When as in Table IV leukaemia at ages 0–4 and 0–14 was examined in the groups of districts with the greatest increases in commuting-*out* (D) separately from commuting-*in* (E), an excess was found only with the latter. No relationship was found with the absolute levels of commuting, either in 1971 (F) or 1981 (G).

The data for ages 0–4 are shown in Table V, analysed both by change in commuting and change in population. Four similar sized groups of person-years were formed from a ranking of districts in terms of commuting increase (the rows) and similarly four groups by population increase 1971 to 1981 (the columns). (If smaller fractions than quarters are used, the proportion of empty cells rises markedly.) A signi-

**Table IV** Adjusted[a] observed to expected ratios of cases of leukaemia in the highest tenth for different measures of commuting and population change (observed numbers in parenthesis)

| | Measure | Period (leukaemias) | Age: 0–4 O/E | Age: 0–4 OBS | Age: 0–14 O/E | Age: 0–14 OBS |
|---|---|---|---|---|---|---|
| A | Total commuting change 1971–81 | 1962–71 | 0.82 | (21) | 0.75 | (32) |
| B | Measure as A[b] | 1972–85 | 2.15[e] | (46) | 1.99[e] | (79) |
| | Excluding Reading[b] | 1972–85 | 2.38[e] | (34) | 1.97[e] | (62) |
| C | Measure as A | 1976–85 | 2.00[e] | (34) | 1.72[e] | (54) |
| | Excluding Reading | 1976–85 | 1.91[d] | (24) | 1.72[d] | (40) |
| D | Commuting-out change 1971–81 | 1972–85 | 1.36 | (34) | 1.25 | (63) |
| E | Commuting-in change 1971–81 | 1972–85 | 1.64[d] | (46) | 1.45[d] | (79) |
| | Excluding Reading | 1972–85 | 1.60[c] | (34) | 1.44[d] | (62) |
| F | Total commuting level 1971 | 1972–85 | 0.94 | (25) | 0.93 | (49) |
| | Excluding Reading | 1972–85 | 0.67 | (13) | 0.83 | (32) |
| G | Total commuting level 1981 | 1972–85 | 0.94 | (25) | 0.93 | (49) |
| | Excluding Reading | 1972–85 | 0.67 | (13) | 0.83 | (32) |
| H | Population change 1971–81 | 1972–85 | 1.40 | (33) | 1.22 | (58) |

[a]Adjusted to the lowest group of the relevant measure as reference. [b]Expected numbers based on county borough specific rates in 1962–71. [c]$P < 0.05$, [d]$P < 0.01$, [e]$P < 0.001$.
Composition of the above (and lowest, reference) groups:
A, B, C – as in Table III.
D – Gloucester, Ipswich, Luton, Exeter (Liverpool).
E – Portsmouth, York, Gloucester, Lincoln, Reading (Liverpool).
F – Nottingham, Reading, Oxford (Torbay, Plymouth, Hastings, Eastbourne).
G – Nottingham, Reading, Oxford (Torbay, Plymouth, Hastings, Eastbourne).
H – Luton, Torbay, Hastings, Lincoln, Eastbourne (Liverpool).

**Table V** Leukaemia at ages 0–4: observed to expected ratios[a] by quarters of population increase and of commuting increase 1971–1981 (Observed numbers in parenthesis)

| Quarters of relative population increase | Quarters of commuting increase: 1 | 2 | 3 | 4 (Highest) |
|---|---|---|---|---|
| 1 | 1.00 (31) | | 0.71 (13) | 0.98 (8) |
| 2 | | 1.18 (23) | 1.30 (24) | 0.89 (16) |
| 3 | 1.27 (33) | 1.03 (18) | 0.92 (14) | 1.23 (13) |
| 4 (Highest) | | 1.06 (24) | 0.88 (10) | 1.77 (45)[b,c] |

[a]Adjusted to the lowest quartile for both measures (Liverpool and Brighton: unadjusted 0.87). [b]Includes Reading, Gloucester, Luton, Lincoln. [c]$P < 0.001$ ($P < 0.01$ after exclusion of Reading). Quarters of population increase: (1) Liverpool, Oxford, Brighton, Portsmouth, Nottingham; (2) Bristol, Bournemouth, Bath, York, Kingston upon Hull, Southampton; (3) Wolverhampton, Stoke, Southend, Grimsby, Blackpool, Exeter, Ipswich, Leicester; (4) Derby, Reading, Plymouth, Gloucester, Luton, Torbay, Hastings, Lincoln, Eastbourne. Quarters of commuting increase: (1) Liverpool, Wolverhampton, Southend on Sea, Brighton, Blackpool; (2) Stoke, Torbay, Hull, Grimsby, Hastings, Bournemouth, Plymouth, Eastbourne; (3) Nottingham, Derby, Bristol, Leicester, Oxford; (4) Southampton, Luton, Portsmouth, York, Bath, Exeter, Ipswich, Lincoln, Gloucester, Reading.

ficant excess of leukaemia is present only in the group of county districts forming the highest category for both measures.

### Discussion

An infective basis for childhood leukaemia is a long-standing hypothesis (Kellett, 1937) and indeed a specific viral cause is established in adult T-cell leukaemia (HTLV 1), as well as in certain animal leukaemias. The absence of marked space-time clustering suggests that, if the disease is infective in origin, leukaemic children cannot be important in spreading the underlying infection, which must mainly be done by symptomless or trivially affected individuals (Smith, 1982). This is obviously a difficult hypothesis to test directly when the relevant agent has not been identified. Increases in population density or situations that in other ways markedly increase the level of social contact will also tend to promote unaccustomed contacts between susceptible and infected individuals, particularly when people come together from a variety of different origins as was the case in rural New Towns during their period of rapid growth. In such situations significant increases of leukaemia at ages 0–4 (Kinlen, 1988; Kinlen *et al.*, 1990) have been found.

Population influxes are far from being the only causes of increased social contact. Wider car ownership, better roads and faster trains now permit many people to live considerable distances from their work, for example in more desirable areas or where housing is less expensive or to avoid moving house. These travel patterns are one of the most striking of modern demographic changes. Information on these patterns is used by central government in defining travel-to-work (employment) areas, which form the basis for determining the allocation of financial aid for new businesses, besides other uses. They have also been the subject of much work by geographers (Lawton, 1968; Hall *et al.*, 1973; Champion *et al.*, 1987, etc). Inevitably therefore much new contact, both direct and indirect, takes place between people from different communities in their areas of work or in the course of commuting. Thus in 1981 the number of people making daily journeys across Oxford's boundaries to reach their work was equivalent to half (50.5%) of that city's population – or alternatively the daily number of such boundary crossings was equivalent to its total population.

In accord with our hypothesis, the present study finds that in 28 county districts leukaemia incidence both at ages 0–14 and 0–4 shows a significant (positive) trend with increasing commuting change. The increase in not steady, however, and only with the most marked commuting increases is there a significant excess of leukaemia, either in individual (Table II) or grouped county districts (Table III). It may be noted that the absence of a steady trend is typical of many infectious diseases in which outbreaks or epidemics only occur when the density, or the numbers, of infected and susceptible individuals in a population reaches some critical point specific for the agent in question.

It was recognised at the outset of this study that if a high level of commuting *were* capable of increasing the incidence of childhood leukaemia, this might well be temporary as in epidemics of most infectious disorders, ending when the number of susceptibles declined below some critical level. Even in 1921 many areas of England and Wales had commuting levels of over 20% (Census of England & Wales, 1927). None of the districts with the highest commuting levels either in 1971 or 1981 had experienced any marked commuting *increases*, and this may be the reason for their not showing any excesses of leukaemia (Table IV, F and G).

When the effects of changes in the commuting-out level (Table IV, D) of the county districts were examined separately from those of commuting-in, only the latter was associated with a significant excess of leukaemia (Table IV, E). However this difference is of uncertain significance since three of the five districts in the highest tenth for 'commuting-*in*' change also belonged to the highest tenth for total commuting increase.

It is noteworthy that the county district with the greatest increase in commuting is Reading. This is also the only district entirely contained within an area in which an excess of childhood leukaemia had already been observed, arousing concern that it might be related to the nearby nuclear establishments in West Berkshire at Aldermaston and Burghfield (Barton *et al.*, 1985; Roman *et al.*, 1987). In fact, Reading children represented about half the child-population of that study area. This comprised those wards with half or more of their area within 10 km of these nuclear sites. This area showed a significant increase at ages 0–14 based on 41 cases, in the same period as our study (1972–85), due to an increase at ages 0–4 (29 observed, 14.42 expected). This excess is not appreciably different in magnitude from the excess found in our study in Reading itself or in group X of total commuting change, excluding Reading. Of the 29 cases aged 0–4, 12 were in Reading and were represented also in our study (O/E ratio 1.74).

It is likely that cases of leukaemia outside Reading but within the West Berkshire excess are also related to commuting increases, for these have occurred markedly throughout this area, much of it lying within so-called 'Silicon Valley'. Boundary changes in 1974 impede examination of such increases in the same way as Reading. Thus substantial amounts of commuting as recorded by the 1971 census would have been concealed had the 1974 boundaries existed, for many journeys would then have occurred within the new area without any boundary crossing. Wokingham district was least affected in this respect since it is composed of only two complete pre-1974 areas, Wokingham MB and RD. The new area experienced a greater commuting increase (10.4%) even more than Reading; it also contributed seven cases of leukaemia at ages 0–4 (expected 3.0) to the excess in the Aldermaston study area. However, no less than 38%, and 51%, of the total commuting in 1971 in what were later Newbury and Basingstoke districts respectively, would have been 'lost' if the 1974 boundaries had been in force (Table VI). Despite this, however, the increases from 1971 to 1981 in commuting level in those enlarged districts as defined in 1974, places them close (9.1 and 7.6%) to Reading, and within the range of group X in the present study (7.2–10.6%). It may be noted that none of the other four county districts in group X are within 10 km (or even 25 km) of a nuclear installation.

The present hypothesis preceded the collection of both the childhood leukaemia and the commuting data, about which we previously knew nothing except for the recently reported excess of the disease in the Aldermaston-Reading area. The relationship therefore seems unlikely to be due to chance or bias. Another possibility however is that the relationship is indirect, reflecting some leukaemogenic factor that is related in some way to increases in commuting. If so, we have been unable to discover it in the census data for the 28 districts we have subsequently examined with respect to social class, changes in social class, and migration. Similarly, we have not found any distinctive changes in the organisation of schools in the group X districts of Table III that might be relevant to the hypothesis about population mixing.

The recent findings imply that a commuting increase equivalent to, say, 10% of a large town's population may produce more effective contacts between infected and susceptible individuals for the postulated infection – than a 10% (or even a 25%) increase in its residential population (Kinlen, 1988; Langford & Bentham, 1990). Large commuting increases with their many direct and indirect opportunities for new contacts and adding to the tides of almost daily ebb and flow may thereby exert more widespread effects than a similar-sized influx of new residents. These suggested effects on the immune response of large populations do not imply that the excess cases of leukaemia in the group X towns will necessarily be in the children of commuters themselves.

The towns in the highest tenth in Table III had not only the greatest commuting *increase* through the 1970s but by 1981 had reached high absolute levels of commuting. The combination of marked increase with a reaching of a high absolute commuting level may therefore be important. The populations of many of the towns studied declined in the 1970s and none showed any marked increase (Table I). Such population changes as there were showed no relationship with leukaemia (Table V, H). However, when leukaemia incidence was analysed by change in commuting level simultaneously with population change, the only category that showed a significant increase was highest for each measure (Table V). The possibility is therefore raised that populations changes, which alone may have little effect, may nevertheless compound the effects of commuting increases. If so, it may be relevant, for example, that several of the areas linked by commuting to the Aldermaston study area have themselves been subject to marked population increases during the study period, of up to 33%. These include the expanding town of Basingstoke, the new town of Bracknell and the large housing development in Earley on the east side of Reading that extends into Wokingham CD.

A significant excess of childhood malignancies other than leukaemia in the period 1971–80 was found in the districts of Reading, Newbury and Basingstoke (COMARE, 1989; Cook-Mozaffari *et al.*, 1987) which include much of the Aldermaston study area. A study has therefore been initiated

**Table VI** Commuting details for county districts at least partly in Aldermaston-Burghfield (A–B) study area and nominal effects of 1974 boundary changes

| *County districts (CD)* | *Number LADs in 1971* | *1971 Commuting and % nominal loss by 1974 boundaries* | | *Commuting change (CD) 1971–81*[b] | *Rank*[a] | *% Children of A–B study area* |
|---|---|---|---|---|---|---|
| Reading | 1 | 40000 | 0% | 9.4% | 27 | 49.6% |
| Wokingham | 2 | 38760 | 13.7% | 10.4% | 22 | 15.5% |
| Newbury | 5 | 48760 | 37.6% | 9.1% | 31 | 23.8% |
| Basingstoke | 3 | 31380 | 51.4% | 7.6% | 53 | 10.8% |
| S. Oxfordshire | 6 | 44840 | 17.0% | 3.9% | 174 | 0.3% |

LAD = Local Authority District Total (In + Out). [a]Among 402 CDs (1 = Highest). [b]As % of 1971 total population. The small part of Wantage Rural District has been ignored.

of the incidence of such malignancies (by subtype), as well as adult leukaemias, in relation to commuting change, as well as of adult leukaemias.

Investigations by the Committee on Medical Aspects of Radiation in the Environment (COMARE, 1989) indicate that the tiny amounts of radiation released into the environment from Aldermaston and Burghfield are too small to cause the increased incidence of leukaemia in their vicinity, which they were unable to explain. However, they regarded a hypothesis about disturbances of herd immunity by population mixed as irrelevant because there had been no sudden population influx into a somewhat isolated area as in the first reported test of the idea (Kinlen, 1988). The present study offers an explanation that is consistent with that hypothesis and with the observations in new towns (Kinlen, 1988; Kinlen *et al.*, 1990). Moreover it is supported by the finding of similar excesses in areas without nuclear installations but which, like the area around Aldermaston and Burghfield, have recently experienced large increases in commuting levels.

A relevant question is whether the prevalence of any infectious disease has also increased in the districts that have experienced the greatest increases of commuting. Published data are limited to only a few infectious diseases, but none show any clear increase in the highest tenth group (data not shown). However, it may be noted that none of those diseases shows a close similarity to the type of disorder to which childhood leukaemia is postulated as belonging.

These findings are consistent with other evidence about the relevance of increases in social contact to the aetiology of childhood leukaemia. As such they represent further support for an infection-based hypothesis for the disease. More specifically they suggest that these contacts can occur (a) between adults, therefore affecting children only indirectly and (b) not only in the home community (Kinlen *et al.*, 1990), but also at work or in travelling to work between residents of different communities. These characteristics are far from unique. Meningococci and the polio viruses are examples of agents that can be transmitted not only among children but also among adults and from adults to children. The present study as well as other work (Kinlen, 1988; Kinlen *et al.*, 1990) suggest that, because of their relevance to herd immunity, population dynamics in their *widest* sense should be considered in communities in which excesses of childhood leukaemia are recorded. In the case of individual-based studies, such effects may not be reliably detected by comparisons of cases with controls drawn from the same community.

We thank Helena Strange for clerical help, David Dipple, Janette Wallis and Fiona O'Brien for computing help and Susan Hill for secretarial assistance. We are also grateful to Dr Paula Cook-Mozaffari and Dr Eve Roman for providing estimated proportions of the children in the Aldermaston and Burghfield study area by county district, and to Dr Robin Mole and Professor David Galton for helpful comments on an earlier draft. We thank the Office of Population of Population Censuses and Surveys, the Information and Statistics Division of the Common Services Agency of the Scottish Health Service, the Registrar General for Scotland, regional cancer registries, clinical trial organisers and the UKCCSG for providing notifications of childhood leukaemia cases. We are grateful to colleagues at the CCRG for their work on the National Registry of Childhood Tumours, in particular Mr M. Loach for computing and Mrs M allen and Dr E.L. Lennox for their part in collecting medical data.

The CRC Cancer Epidemiology Research Group is entirely funded by the Cancer Research Campaign from which LJK holds a Gibb Fellowship. The Childhood Cancer Research Group is supported by the Department of Health and the Scottish Home and Health Department.

### Addendum

A correction for multiple comparisons, omitted from Table III changes the significance levels, so that $P<0.001$ becomes $P<0.01$ and $P<0.01$ becomes $P<0.05$.

### References

BARTON, C.J., ROMAN, E., RYDER, H.M. & WATSON, A. (1985). Childhood leukaemia in West Berkshire. *Lancet*, **ii**, 1248.

CENSUS OF ENGLAND AND WALES 1921 (1927). *General Report*. Part XI. Workplaces. HMSO: London.

CHAMPION, A.G., GREEN, A.E., OWEN, D.W., ELLIN, D.J. & COOMBES, M.G. (1987). *Changing places*. Edward Arnold: London.

COMMITTEE ON MEDICAL ASPECTS OF RADIATION IN THE ENVIRONMENT (COMARE) (1989). *Third Report: Report on the incidence of childhood cancer in West Berkshire and North Hampshire area, in which are situated the Atomics Weapons Research Establishment, Aldermaston and the Royal Ordnance Factory, Burghfield*. Chairman: Professor M. Bobrow. HMSO: London.

COOK-MOZAFFARI, P.J., ASHWOOD, F.L., VINCENT, T., FORMAN, D. & ALDERSON, M. (1987). Cancer incidence and mortality in the vicinity of nuclear installation. England and Wales, 1959–80. Office of Population Censuses and Surveys. *Studies on Medical and Population Subjects*, No. 51, HMSO: London.

HALL, P., THOMAS, R., GRACEY, H. & DREWETT, R. (1973). *The containment of urban England: 1. Urban and metropolitan growth processes or Megalopolis denied*. Allen & Unwin: London.

KELLETT, C.E. (1937). Acute leukaemia in one of identical twins. *Arch Dis. Child.*, **12**, 239.

KINLEN, L. (1988). Evidence for an infective cause of childhood leukaemia: comparison of a Scottish New Town with Nuclear Reprocessing Sites in Britain. *Lancet*, **ii**, 1323.

KINLEN, L.J., CLARKE, K. & HUDSON, C. (1990). Evidence from population mixing in British New Towns 1946–85 of an infective basis for childhood leukaemia. *Lancet*, 577.

LANGFORD, I. & BENTHAM, G. (1990). Infectious aetiology of childhood leukaemia (letter). *Lancet*, 945.

LAWTON, R. (1968). The journey to work in Britain: some trends and problems. *Reg. Stud.*, **2**, 27.

ROMAN, E., BERAL, V., CARPENTER, L. & 4 others (1987). Childhood leukaemia in the West Berkshire and Basingstoke and North Hampshire District Health Authorities in relation to nuclear establishments in the vicinity. *Br. Med. J.*, **294**, 597.

SMITH, P.G. (1982). Spatial and temporal clustering. In *Cancer Epidemiology and Prevention*. Schottenfield, D. & Fraumeni, J.R. (eds). Philadelphia: W.B. Saunders.

STILLER, C.A. (1985). Descriptive epidemiology of childhood leukaemia and lymphoma in Great Britain. *Leuk. Res.*, **6**, 671.